# Unintentional non-adherence: can a spoon full of resilience help the medicine go down?

**DOI:** 10.1136/bmjqs-2013-002276

**Published:** 2013-09-16

**Authors:** Dominic Furniss, Nick Barber, Imogen Lyons, Lina Eliasson, Ann Blandford

**Affiliations:** 1 UCL Interaction Centre (UCLIC), University College London, London, UK; 2 Department of Practice and Policy, Centre for Medication Safety and Service Quality, UCL School of Pharmacy, London, UK; 3 Faculty of Medicine, Centre for Haematology (Honorary Research Associate), Imperial College London, London, UK; 4 Atlantis Healthcare (Clinical Strategist), London, UK

**Keywords:** Human Error, Medication Safety, Human Factors, Compliance

## INTRODUCTION

Non-adherence to medication is a ‘worldwide problem of striking magnitude’.^1^ It has consequences for the health of patients and is a great concern for healthcare providers in terms of patient outcomes and healthcare costs. Theoretical research has mainly focused on intentional non-adherence: for example, when people choose not to take their medication. However, unintentional non-adherence also accounts for a significant proportion of the problem: when people mean to take their medication in the right way but do not. This area is under-researched. In this paper, we bring a new perspective to this problem by exploring what contribution ‘resilience’ could make to it. Resilience engineering focuses on a system's ability to maintain performance, avoid error, compensate for poor circumstances and cope with disturbances. So, rather than focus on how things go wrong, we propose to exploit and enhance how things go right.

## Non-adherence

The National Health Service in England processed 962 million prescriptions in 2011, which had an ingredient cost of £8.8 billion,^2^ and it is estimated that 30%–50% of prescribed medication are not taken correctly.^3^ A report prepared for a summit of European health ministers in 2012 estimated that non-adherence contributes approximately 57% of $500 billion total avoidable costs attributed to suboptimal medicine use globally each year.^4^ Horne *et al*^3^ provide a broad and comprehensive review of the research in this area. Their report shows that the problem is complex and multifaceted, and they call for more research to tackle this important issue. They argue that improving the effectiveness of adherence interventions could have a greater impact on public health than improvements in specific medical treatments.^3^

Haynes *et al*^5^ note that current successful interventions for non-adherence are multifaceted, complex, labour intensive and at best have modest effects; they call for innovative approaches to assist patients to follow prescriptions for medications. To help gain traction on this problem, different theoretical frameworks have been applied. Barber *et al*^6^ point out that the most commonly used explanations of non-adherence have their roots in psychology: for example, social cognition models such as the theory of reasoned action and the self-regulatory model. However, these give little insight into unintentional non-adherence.

Barber^7^ and Barber *et al*^6^ have found theoretical leverage for understanding unintentional non-adherence by applying the human error literature to their data. Reason^8^ distinguishes between three broad categories of error that have different cognitive causes: slips and lapses, mistakes, and violations. Slips and lapses occur when someone has a momentary lapse of attention and forgets to do something or does the wrong thing in a routine procedure despite knowing what to do; mistakes are the misapplication of knowledge so that people make the wrong decision about what to do; and a violation is where people have the correct knowledge but intentionally choose to break rules. Barber *et al*^6^ applied this theory to data about instances of non-adherence and found that it provided a way of seeing different patterns, problems and solutions in the situation. First and foremost, it provided a structure for analysing instances of non-adherence in terms of their cognitive causes. This was particularly useful for gaining insight into causes of unintentional non-adherence, which are largely neglected in the literature. Second, it moved blame away from the patient to investigate the contribution of the wider system in cases where someone did not adhere. These include system level factors that are not purely cognitive, such as poor communication from medical staff, the patient's financial situation, their life style, poor labelling on medication and confusing instructions.

## A resilience perspective

Efforts to improve quality and safety in healthcare tend to focus on when things go wrong, for example, ‘to err is human’.^9^ However, this can be to the detriment of noticing the informal and interesting ways in which systems go right despite adversity.

Resilience engineering is a relatively new research field that focuses on the positive aspects of resilient systems:^10^ how systems anticipate, monitor, respond and learn so that they are able to avoid and recover from error and maintain successful performance.^11^ This perspective has been applied to emergency scenarios where systems have faced an extreme situation that they have not had to cope with before, and it has been applied to everyday aspects of system performance that keep a system working well. It has been applied to a wide variety of contexts within healthcare, including emergency departments,^12^ handover^13^ and surgery.^14^ In studies of programming infusion pumps, Furniss *et al*^15^ found that nurses organise their work in intelligent ways to improve their performance and to reduce the risk of making errors. Methodologically, it is important not just to hold errors and incidents up to scrutiny, but also to examine ‘normal’ practice to identify the intelligent ways in which things go right despite poor behaviour, poor design, poor systems and poor circumstances.^16^

One area of theoretical and practical interest related to this work is the recognition and development of ‘resilience strategies’ which can help people avoid error and improve performance.^16^ ^17^ These include creating cues to remember things, separating similar objects and tasks so that there is less likelihood of confusion, and checking you have got the correct resources before committing to an action. These strategies provide a new perspective on problems associated with adherence.

## A change of perspective for non-adherence

Positive behaviours related to adherence have been noted in other studies, but they have not had the strength of theoretical motivation and focus we propose here. For example, Barber *et al*^6^ refer to the strategies that patients employ to help them adhere by adjusting routines and creating reminders, and Eliasson^18^ reported many strategies to aid adherence in patients prescribed imatinib (a life-saving drug for chronic myeloid leukaemia), such as taking medication with food, crushing tablets and dissolving them in water, using a TV programme and family members as a prompt, and using a visual prompt in the bathroom or kitchen. These strategies help the patients fit imatinib into their daily routine, use prompts to help them remember to take their medication and find ways of coping with side effects, which can all influence whether they adhere or not.^19^ These strategies help people to better cope with the challenges of adherence and reduce the likelihood of non-adherence, particularly if unintentional. Although we have dichotomised forms of non-adherence in this paper, as is common, they interact, which forms part of the story of resilience and adherence. For example, weak intent can lead some not to create strategies to guard against unintentional difficulties to ensure they take the drug reliably; in contrast, constant unintentional difficulties in managing to take a drug can lead to dwindling intent, particularly if there are no overt consequences of non-adherence. We focus in this paper on unintentional non-adherence because it is a significant problem that is under-theorised.

A resilience perspective has concepts and frameworks that can be used to better explore unintentional non-adherence. For example, Furniss *et al*^17^ identify abstract categories of resilience strategies that can be used to recognise and identify resilience more broadly. Table 1 shows these abstract categories of resilience with examples, which illustrate their application to medication taking/adherence.

**Table 1:** Resilience strategies with examples for adherence (adapted from Furniss *et al*^17^)

Resilience strategy	Definition	Example
Cue creation to support prospective memory	A cue is created as a reminder about something in the future	Setting an alarm to remember to take medication at a particular time
Premature-completion awareness	Action is taken as a reminder about ‘X’ after the main goal has been achieved, where ‘X’ is normally a secondary task	Leaving used and empty medication packaging out, rather than putting it straight into the bin, as a reminder to order more if it is needed
Pre-emptive separation and disambiguation	Things are separated or differentiated so they are not mixed up	Moving similar looking pills into monitored dosing boxes or labelling them in a different and salient way
Precommitment check	Things are checked before committing to a course of action	Making sure all the parts for home nebulisation of drugs are present before starting the procedure
Managing resource availability	Resources are managed so they are available for action	Having medication at work and at home just in case it is forgotten at one location
Routine adjustment	Routine is adjusted in response to a threat or opportunity	Adjusting time of taking medicines when travelling between time zones
Safety reinforcement	Where some safety barrier, procedure or practice is reinforced	Double checking blood glucose levels and insulin dosage before injecting

Some of the strategies in table 1 are observed in Eliasson;^18^ for example, using an alarm on a watch is an example of cue creation; taking medication as part of a set morning routine and avoiding the evening which could be more unpredictable and more prone to forgetting is one example of routine adjustment; and carrying around medication so that it is available wherever the patient may be is an example of managing resource availability. These categories allow one to reflect on strategies at a more abstract level. They provide a focus and a vocabulary so we can talk about these behaviours collectively and in new ways.

Further research needs to explore the quality of individual strategies. For example, Furniss *et al*^17^ note that the quality of a cue depends on whether the cue is salient, linked to the thing to be recalled and task related. We might speculate that using a TV programme as a cue is vulnerable to failure because it is indirectly linked to taking medication, watching the programme is not task related and it depends on watching the TV. In contrast, using a mobile phone alarm as a cue could specify which medication to take, it can be snoozed until the medication has actually been taken so it integrates with the task more directly and it can be with the person while on the move.

Further research also needs to develop resilience theory. Eliasson^18^ reports problems and strategies that do not fit into table 1. For example, some patients reported difficulties in remembering whether they had taken their medication or not, which could lead them to take it again and overdosing (a form of non-adherence), or lead them to missing a dose for fear of taking it twice. This is an issue for retrospective memory that does not appear in table 1. Furniss *et al*^17^ note that the concepts they propose still require further research and development. Empirical observations such as these can be used to suggest new resilience strategies and in doing so can contribute to the development of this theory, as well as the theory contributing to improving adherence.

In addition to the categorisation of resilience strategies, the resilience markers framework^16^ can support understanding of resilience in practice by helping to describe how adherence takes place. Particularly, how performance is maintained in the rich varieties of ‘normal’ and more extreme contexts in which people try to adhere, and to include the effects of their illness, type of treatment, emotional stress, cognitive impairments, and patients’ social and financial situations. By focusing on these and other system level factors, we widen the possible explanations for adherence and non-adherence. The resilience markers framework elaborates how individuals in different contexts can use similar strategies, what dependencies might influence the use of different strategies and what difficulties they might be trying to overcome.

Briefly, at the heart of the framework is a *resilience repertoire* which is a ‘library’ of strategies that can be adapted and deployed in different contexts; this would be something similar to the examples in table 1. The use of these strategies will depend on whether the person has the *resources* to use them, for example, you cannot set a phone alarm without a phone. It will also be influenced by the *mode of operation* where certain classes of behaviour will have an impact on the vulnerabilities of the system and the strategies that can be used. For example, if a patient is illiterate then strategies that involve reading will not help, and if a patient is alone then strategies that involve help from friends and family cannot help. More positively, if patients have a poor short term memory then developing support for this might be particularly helpful, and if they are comfortable with new technology then developing a strategy that involves this could contribute to a successful outcome.

The reasons for non-adherence are diverse and the strategies for tackling it need to be equally diverse, being tailored to individual patients and their circumstances. Patients, carers and clinical staff could create a library of strategies for broader populations of patients, carers and clinicians to adopt and adapt to support adherence. This resonates with the suggestion of Vermeire *et al*^20^ of a menu of compliance-enhancing strategies from which an appropriate strategy could be selected for individual patients and their treatment, but gives further detail and a theoretical basis to move forward from.

## Conclusions

Resilience is more than just the avoidance of error; it can exploit and enhance successful performance in its own right. For adherence, a resilience perspective is a new approach that focuses on enhancing strategies to cope with adherence and reducing the likelihood of unintentional non-adherence. We have seen that episodes of resilient behaviour can be identified and reflected upon through more abstract categories of resilience strategies. We have also seen that understanding the variability of situations in which they are used can be supported by using the resilience markers framework. We need to understand the resilience strategies that patients develop, the best ways of sharing these strategies and testing whether other patients adopt them, adapt them and find them useful for successfully adhering to their own medication regimes. These efforts could work towards developing a library of resilience strategies that are pertinent to different communities of patients, carers and clinicians for use in different circumstances so that more people are able to manage their medications effectively.
